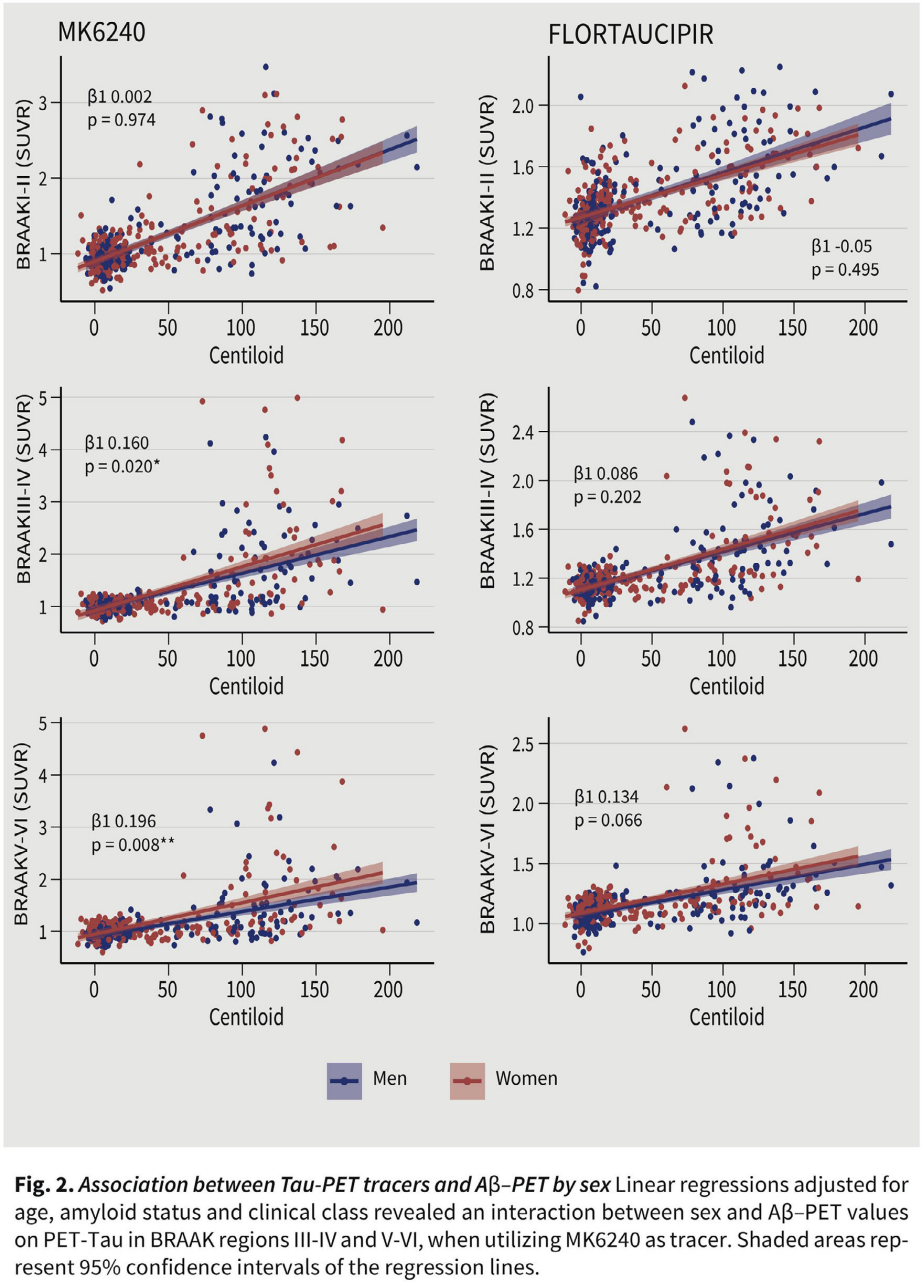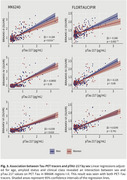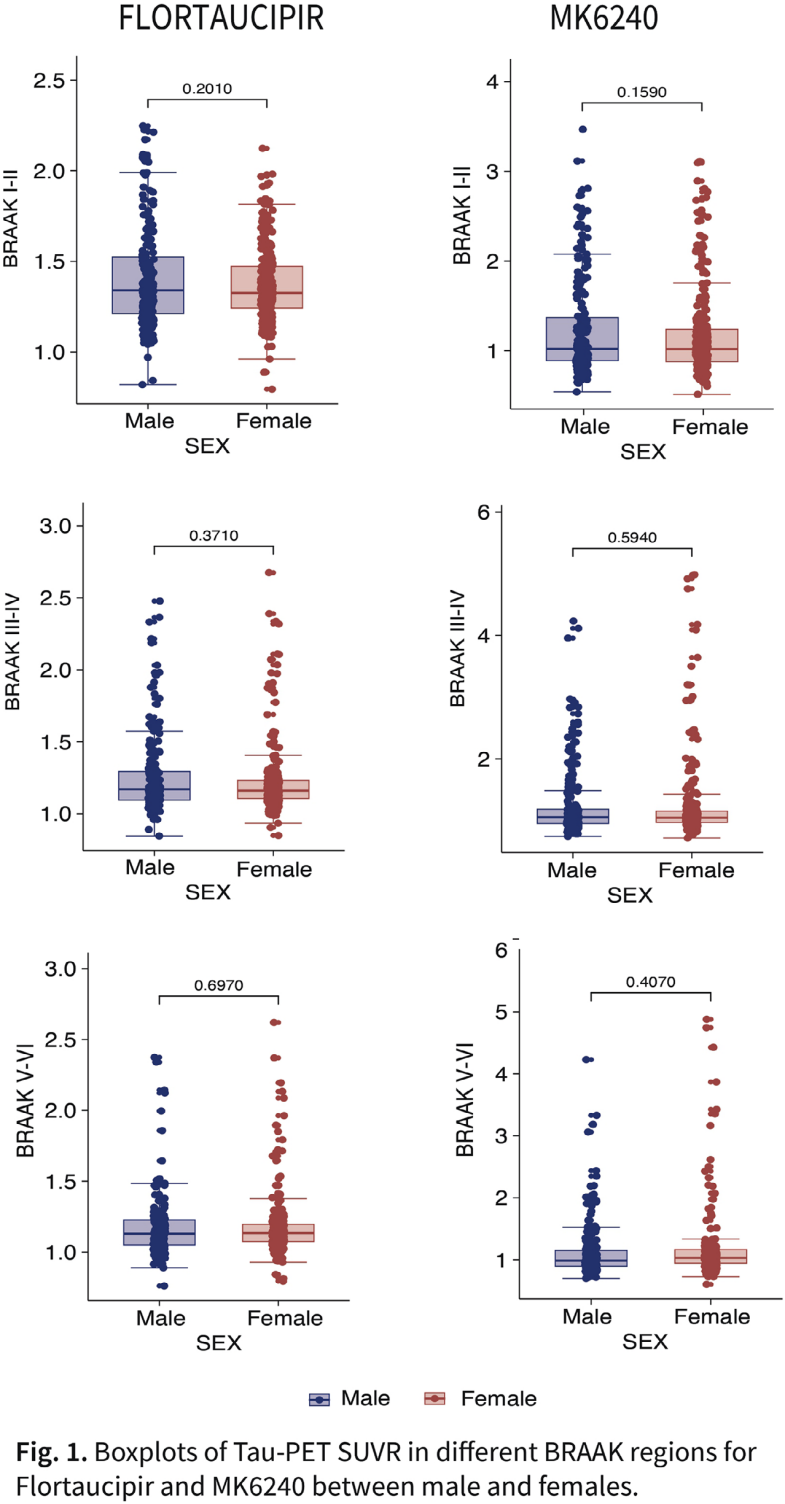# The Impact of Sex in Tau‐related AD pathology

**DOI:** 10.1002/alz70856_106862

**Published:** 2026-01-08

**Authors:** Lorenza P. Botton, Bruna Bellaver, Guilherme Povala, Pamela C.L. Ferreira, Guilherme Bauer‐Negrini, Firoza Z Lussier, Livia Amaral, Joseph C. Masdeu, Dana L Tudorascu, Thomas K Karikari, David N. Soleimani‐Meigooni, Juan Fortea, Val J Lowe, Hwamee Oh, Belen Pascual, Brian A. Gordon, Pedro Rosa‐Neto, Jess Baker, Tharick A Pascoal

**Affiliations:** ^1^ School of Medicine, Pontifícia Universidade Católica do Rio Grande do Sul (PUCRS), Porto Alegre, Rio Grande do Sul, Brazil; ^2^ University of Pittsburgh, Pittsburgh, PA, USA; ^3^ Houston Methodist Research Institute, Houston, TX, USA; ^4^ UCSF Alzheimer's Disease Research Center, San Francisco, CA, USA; ^5^ Sant Pau Memory Unit, Hospital de la Santa Creu i Sant Pau, Biomedical Research Institute Sant Pau, Barcelona, Spain; ^6^ Mayo Clinic, Rochester, MN, USA; ^7^ Brown University, Providence, RI, USA; ^8^ Washington University School of Medicine, St. Louis, MO, USA; ^9^ McConnell Brain Imaging Centre, Montreal Neurological Institute, McGill University, Montreal, QC, Canada; ^10^ University of New South Wales, Sydney, Australia; ^11^ University of Pittsburgh School of Medicine, Pittsburgh, PA, USA

## Abstract

**Background:**

Studies suggest that sex influences the deposition of tau in the human brain and impacts on the relationship of tau with AD‐related outcomes. The aim of this study is to evaluate the extent to which sex influences PET‐Tau related outcomes and whether this effect is related to specific tau PET tracers.

**Method:**

We assessed 456 participants from the HEAD study (mean age = 66.07 ± 13.05). All individuals had available Tau‐PET with Flortaucipir and MK6240, Aβ‐PET and a subset had plasma *p*‐Tau 217 (*n* = 352). We conducted a linear regression analysis between sex and PET Tau to evaluate the direct influence of sex on PET Tau SUVR. Next, we assessed whether sex affected the relationship between Tau PET (in BRAAK I‐II, III‐IV, V‐VI regions) and Aβ‐PET or plasma *p*‐Tau 217 by adding an interaction term in the associations. All analyses were corrected by age, clinical classification, and amyloid burden.

**Result:**

No significant differences were observed between male and female SUVR levels in either Flortaucipir and MK6240 tracers (Figure 1). However, when assessing the relationship between Aβ‐PET and Tau, a statistically significant interaction with sex was seen between MK6240 in BRAAK III‐IV (β = 0.160, *p* = 0.020 ; Figure 2) and BRAAK V‐VI regions (β = 0.196, *p* = 0.008; Figure 2), with women having a stronger association. Furthermore, we found an interaction between sex and *p*‐tau217 on Tau PET SUVR in BRAAK regions I‐II using MK6240 or Flortaucipir (β = ‐0.184, *p* =  0.014; β = ‐0.266, *p* =  0.002; Figure 3) indicating that women present a weaker relationship between *p*‐tau217 and Tau PET.

**Conclusion:**

Our results did not show any differences between men and women in Tau‐PET SUVR uptake across tau tracers. However, we found that sex affected how Aβ‐PET and plasma *p*‐tau217 were associated with Tau‐PET uptake. Further studies are needed to elucidate the underpinnings of the link between sex and the association between these biomarkers.